# Modified Nano-Montmorillonite and Monensin Modulate *In Vitro* Ruminal Fermentation, Nutrient Degradability, and Methanogenesis Differently

**DOI:** 10.3390/ani11103005

**Published:** 2021-10-19

**Authors:** Yosra Soltan, Amr Morsy, Nesrein Hashem, Mahmoud Elazab, Mohamed Sultan, Haneen Marey, Gomaa Abo El Lail, Nagwa El-Desoky, Nourhan Hosny, Ahmed Mahdy, Elsayed Hafez, Sobhy Sallam

**Affiliations:** 1 Animal and Fish Production Department, Faculty of Agriculture, Alexandria University, Alexandria 21545, Egypt; nesreen.hashem@alexu.edu.eg (N.H.); nh9101282@gmail.com (H.M.); enagwa278@gmail.com (N.E.-D.); s_sallam@yahoo.com (S.S.); 2 Livestock Research Department, Arid Lands Cultivation Research Institute, City of Scientific Research and Technological Applications, Alexandria 21934, Egypt; amrsalah277@hotmail.com (A.M.); melazab@srtacity.sci.eg (M.E.); nourhansaad80@yahoo.com (N.H.); 3 Economic and Agribusiness Department, Faculty of Agriculture, Alexandria University, Alexandria 21545, Egypt; m.sultan2270@yahoo.com; 4 Soil and Water Sciences Department, Faculty of Agriculture, Alexandria University, Alexandria 21545, Egypt; gelsayh@yahoo.com (G.A.E.L.); amahdy73@yahoo.com (A.M.); 5 Plant Protection and Biomolecular Diagnosis Department, Arid Lands Cultivation Research Institute, City of Scientific Research and Technological Applications, Alexandria 21934, Egypt; elsayed_hafez@yahoo.com

**Keywords:** clays, nanoparticles, methane, degradability, cation-exchange capacity, surfactants

## Abstract

**Simple Summary:**

Natural montmorillonite (NM) is the most common clay used as a feed additive in ruminant diets. Under normal pH conditions, it can adsorb hydrogen and may affect methane (CH_4_) formation; however, it possesses less efficiency than other clays. Due to NM’s negative charge flat surface and positive charge edges, its physicochemical properties can be modified by cationic or anionic surfactants. Therefore, two types of modified nano-montmorillonite (MNM) were developed by ion-exchange reactions using cationic and anionic surfactants. Comparisons were made with monensin as a rumen modulator to reduce CH_4_ emission from ruminants. The results indicated that the physicochemical properties of both MNM types were enhanced (e.g., cation-exchange capacity and zeta potential). All MNM clays and monensin successfully reduced rumen CH_4_ production and ammonia concentration; however, clay modified by cationic surfactant was more efficient than what was modified by anionic surfactant in modulating *in vitro* rumen fermentation properties

**Abstract:**

Two types of modified nano-montmorillonite (MNM) were developed by ion-exchange reactions using two different surfactants; sodium dodecyl sulfate (SDS) and cetyltrimethylammonium bromide (CETAB), to prepare MNM_SDS_ and MNM_CETAB_, respectively. Both MNM types were on the nano-scale and had higher cation-exchange capacity values than NM clay. The MNM_CETAB_ had the highest zeta potential (−27 mV) compared with the other clays. Effects of MNM types on *in vitro* ruminal batch culture fermentation, nutrient degradability, and methane (CH_4_) emission compared with monensin were evaluated *in vitro* using a semi-automatic gas production system. The experimental treatments were the control (0 supplementations), monensin (40 mg/kg DM), and NM (5 g NM/kg DM), and two levels of MNM_SDS_ and MNM_CETAB_ were supplemented at 0.05 (low) and 0.5 (high) g/kg DM to the control basal feed substrate. Among the experimental treatments, the high dose of both MNM types reduced (*p* < 0.01) CH_4_ production and ammonia concentrations compared with the control, while only MNM_CETAB_ treatment tended to increase (*p* = 0.08) the truly degraded organic matter compared with monensin. All MNM treatments increased (*p* < 0.01) acetate molar proportions compared with monensin. The high MNM_CETAB_ increased (*p* < 0.01) the *in vitro* ruminal batch culture pH compared with the control and monensin. The MNM_CETAB_ supplemented at 0.5 g/kg DM is the most efficient additive to reduce CH_4_ emission with the advantage of enhancing the *in vitro* nutrient degradability of the experimental feed substrate. These results indicated that MNM could modulate the *in vitro* ruminal fermentation pattern in a dose- and type-dependent manner.

## 1. Introduction

Enteric fermentation is considered an actual cause of climate change and environmental pollution due to the emissions of greenhouse gases (e.g., methane (CH_4_) and carbon dioxide (CO_2_)) [1]. Methane is 28 times more powerful as a potent greenhouse gas than CO_2_; it is directly produced by ruminal methanogens, while other hydrogen-producing microorganisms (e.g., *protozoa*) can indirectly contribute the CH_4_ formation through a synergistic association relationship with methanogens [1,2,3]. Moreover, the CH_4_ emission from ruminants represents a significant loss of dietary energy, which could be redirected towards valuable animal products [2]. Therefore, various rumen fermentation modifiers have been applied for ruminants to inhibit CH_4_ emission; however, the dietary antibiotic ionophores (e.g., sodium monensin) seem to be the most successful ones [3]. Ionophores are polyether antibiotics acting as inhibitors to deamination and H+ producing bacteria. They mainly disturb the bacterial cell wall membrane through ion exchange capacity, specifically H+/Na+ and H+/K+ antiport activity [1]. Nevertheless, the current global scenario has shifted the interest towards natural and safe feed supplements instead of antibiotics for protecting the environment and producing safe animal products [2,3].

Geophagy (eating clays) is a common natural habit of ruminants. Therefore, several clay classes are recognized as safe for animal and human consumption [4,5]. Natural montmorillonite (NM), also named microcrystalline kaolinite, has an advantage over other clays because of its high availability, low cost, large surface area, small particle size, and high ion exchange activity characteristics [5,6]. Moreover, it acts as a buffering agent to regulate acidosis. Additionally, it works against bloat and diarrhea and can adsorb heavy metals and aflatoxins [7]; therefore, NM was widely used as a feed additive for ruminants. Natural montmorillonite has lower antibacterial effects than other nano or organically modified clays [5,6]. Tate et al. [5] reported the first investigation on using NM as a rumen modifier to reduce rumen CH_4_ production *in vitro* through a direct inhibition effect on methanogens. They found that NM was less effective in inhibiting methanogenesis than other kaolinite clays.

Montmorillonite platelets have a unique ionic composition: a negative charge flat surface and positive charge edges [5,6]. Thus, inorganic ions in NM can be effectively exchanged with both cationic and anionic surfactants through ion exchange reactions [8]. This phenomenon was exploited to modify and enhance the cation exchange capacity (CEC) and antibacterial properties of NM. Compared with NM, modification of montmorillonite using cationic surfactants (e.g., quaternary ammonium salts) leads to damage to the cellular membrane of Gram-positive bacteria cells (e.g., *Staphylococcus aureus*) [6]. In addition, montmorillonite modified by anionic organosulfur surfactants that have antimicrobial properties (e.g., sodium dodecyl sulfate (SDS)) possesses high CEC, which in turn increased the uptake of heavy metal ions [8]. Additionally, modifying NM by mechanical grinding improved the clay’s stability and physicochemical properties while exhibited intense antibacterial activity against *Escherichia coli* [4].

Recently, grinding the natural clays (e.g., zeolite) in the nano-scale (1–100 nm) enhanced the clay’s chemical stability and physicochemical properties [9]. At the same time, it reduced CH_4_ and ammonia production while improved the fiber or organic matter rumen degradability in a dose and particle-size-dependent manner [9]. It can be speculated that, if nano-scale dispersion for modified montmorillonite would be achieved, new exceptional physicochemical properties might appear for the modified clays and/or the lowest effective supplementation dose could be obtained. Our working hypotheses are as follows: (1) The actively modified nano montmorillonite (MNM) can be prepared by chemical and nano grinding modifications. (2) The prepared MNM can modulate ruminal *in vitro* batch culture fermentation patterns, including methanogenesis. Therefore, we developed two different types of MNM using anionic (sodium dodecyl sulfate (SDS)) and cationic (cetyltrimethylammonium bromide (CETAB)) surfactants. This study is the first investigation to evaluate the effects of modified clays compared with antibiotic monensin on *in vitro* fermentation, protozoal count, and nutrient degradability.

## 2. Materials and Methods

This study was carried out at the Advanced Laboratory of Animal Nutrition, Faculty of Agriculture, Alexandria University, Alexandria, Egypt. All procedures and experimental protocols were carried out according to the guidelines for the care and use of animals in research of Alexandria University (AU 08-200415164).

### 2.1. Preparation of MNM Products

Natural montmorillonite clay (NM) was commercially supplied (Egypt Bentonite and Derivatives Co., Alexandria, Egypt) in powder form. The NM clay contained 544 g/kg dry matter (DM) of silicon dioxide, 190 g/kg dry matter of aluminum oxide, 135 g/kg dry matter of Iron(II) + iron(II, III), 52 g/kg dry matter of sodium oxide, 18.1 g/kg dry matter of titanium dioxide, 31 g/kg dry matter of magnesium oxide, 16 g/kg dry matter of calcium oxide, 12 g/kg dry matter of potassium oxide, and 1.9 g/kg dry matter of phosphorus pentoxide. The method of Bujdáková et al. [10] was followed to obtain the experimental MNM types, using two different surfactants, SDS and CETAB (Sigma Aldrich Co., Irvine, Scotland), to prepare the modified nano montmorillonite by SDS (MNM_SDS_) and the modified nano montmorillonite by CETAB (MNM_CETAB_), respectively. To obtain the nano-scale of MNM_CETAB_ and MNMsds, the resulting materials were ground using High-Energy Laboratory Planetary Ball Miller (Retsch PM, VERDER SCIENTIFIC, North Rhine-Westphalia, Haan, Germany) for six hours with a reverse rotation speed of 300 rpm and vial rotation speed of 600 rpm with the ball to powder ratio of 9:1 mass/mass.

### 2.2. Physicochemical Properties of NM, MNMsds, and MNM_CETAB_

The particle size and the surface charge of the experimental clays were measured by zeta potential analysis using a Malvern ZETASIZER Nano series (Malvern, Worcestershire, United Kingdom) with a range of particle size detection from 0.3 nm to 10 microns at 25.0 ± 1 °C temperature, count rate (kcps) 347.4, measurement position (mm) 2.0, and attenuator 7.0.

The pH and electrical conductivity (EC) of the experimental feed additives were determined in a suspension of clay and distilled water (pH = 6.80) in a ratio of 1:2.5 by a multi-parameter pH meter (GLP 21 model; CRISON, Barcelona, Spain). The cation-exchange capacity was measured according to [11] using 1 M sodium acetate−0.1 M sodium chloride.

The transmission electron microscope (TEM) was used to provide dimensional images for the experimental NM, MNMsds, MNM_CETAB_ feed additives to detect the size and shape of their nanoparticles. Clay samples were coated with gold to improve the imaging of the clay sample and scanned using a TEM (JSM1400 plus-JEOL, Los Angeles, CA, USA) operated at a vacuum of the order of 10, and the accelerating voltage of the microscope was kept in the range of 10–20 kV.

The functional groups of the experimental feed additives were identified by Fourier Transform Infra-Red Spectroscopy (FTIR) using an infrared spectrometer (Shimadzu FTIR-8400S, Osaka, Japan) equipped with a deuterated triglycine sulfate (DTGS) KBr detector and purge gas generator.

### 2.3. In Vitro Gas Production (GP)

#### 2.3.1. Basal Feed Substrate and Experimental Design

A basal feed substrate consisted of 500 g/kg dry matter of concentrate and 500 g g/kg dry matter of berseem hay (*Trifolium alexandrinum*) of the 3rd cut; this feed substrate was formulated to fulfill the national research council [12] nutrient requirements of growing cattle. The basal feed substrate was chemically analyzed according to the Association of Official Analytical Chemists [13] for DM, organic matter (OM), crude protein (CP; as 6.25 × nitrogen), and ether extract (EE). Neutral detergent fiber (NDF) and acid detergent fiber (ADF), and lignin were analyzed according to Van Soest et al. [14]. All plant cell well fractions were sequentially determined using the semi-automatic fiber analyzer (ANKOM, model A2001, Macedon, New York, NY, USA) using the same sample in a filter bag (F57-ANKOM Technology Corporation, Macedon, New York, NY, USA). Primary ingredients and chemical analyses of the basal feed substrate are shown in Table 1.

The experimental treatments were the control (basal feed substrate without supplementations), monensin (basal feed substrate supplemented with 40 mg/kg DM sodium monensin (Rumensin^®^, Elanco, Itapira, São Paulo, Brazil)), and NM (basal feed substrate supplemented with 5 g NM/kg DM), and four MNM treatments were tested using two doses (low and high) supplemented to the basal feed substrate. The low dose was 0.05 g MNM_SDS_ or MNM_CETAB_/kg DM, and the high dose was 0.5 g MNM_SDS_ or MNM_CETAB_/kg DM. The experimental dose of NM was tested according to Maki et al. [7]. Monensin was evaluated because it is one of the most common ionophore feed additives used to reduce ammonia and CH_4_ emissions [1,3]. The experimental dose of monensin was the manufacturer’s recommendation; this dosage (with the same source) was previously found to decrease CH_4_ production and ammonia concentration *in vitro* while exerting minimal effects on the *in vitro* degradation of OM and total short-chain fatty acids (SCFAs) concentrations [3]. Therefore, a dose of 40 mg/ kg DM of monensin was used in the current study.

#### 2.3.2. Procedure of GP

The experimental treatments were evaluated using the semi-automatic GP system according to Bueno et al. [15] and adapted to Soltan et al. [3]. The ruminal contents were collected separately from three fasted, slaughtered crossbred cow calves (440 ± 5 SE kg body weight) from the slaughterhouse that belongs to the Department of Animal and Fish Production, Faculty of Agriculture, Alexandria University, to avoid any unusual individual rumen environmental conditions [16]. These slaughtered calves were fed a local diet for beef production consisting of 500 g berseem hay (*Trifolium alexandrinum*) and 500 g commercial concentrate mixture (145 g crude protein/kg DM) ad libitum. The ruminal contents were transferred into pre-warmed thermo-containers (40 °C) under CO_2_ flushing. To prepare the ruminal inocula (*n* = 3) to the *in vitro* incubation, the ruminal contents of each calf were blended for 10 s, squeezed by four layers of cheesecloth, and kept in water bath (39 °C) under continuous flushing of CO_2_.

For each ruminal inoculum, six *in vitro* incubation flasks (Arab Pharmaceutical Glass Company S.A.E., Suez, Egypt) as analytical replicates were prepared for each experimental treatment in addition to blank flasks (containing Menke’s buffered medium and ruminal inoculum) [15] that were used to obtain the net gas production values and internal standard flasks (containing Menke’s buffered medium, ruminal inoculum, and berseem clover hay) to correct for sensitivity variations induced by the inocula; variations above 10% were rejected [16].

A sample of 500 mg of each experimental feed substrate was accurately weighed into an incubation flask and incubated with 30 mL of the buffer solution and 15 mL of the inoculum while leaving a headspace of 75 mL [3,16]. The flasks were closed with 20 mm butyl rubber stoppers, sealed with aluminum seals, and incubated at 39 °C in a forced air incubator (FLAC STF-N 52 Lt, Treviglio, Lombardy, Italy) for 24 h. The headspace gas pressure of the flask was determined at 3, 6, 9, 12, and 24 h from the incubation start using a pressure transducer and a data logger (Pressure Press Data GN200, Piracicaba, Sao Paulo, Brazil). The volume of GP (mL) was calculated as 4.97 × measured pressure (psi) + 0.171 (*n* = 500; r^2^ = 0.99) [3].

For CH_4_ determination, one mL of the headspace gas was sampled at each pressure measuring time by a 3 mL syringe (Dawliaico, Assiut, Egypt) and was accumulated in 5 mL vacutainer tubes (BD Vacutainer^®^ Tubes, Jersey, NJ, USA). Methane concentrations were determined by gas chromatography (GC, Model 2014, Drawell Scientific Instrument Co., Ltd., Shanghai, China) equipped with a Molesieve 5A micro packed column (1 m, 2 mm ID, Ref no. 80440-800; Restek, Bellefonte, PA, USA). The GC separation conditions were reported in detail by Sabry et al. [17].

#### 2.3.3. In Vitro Ruminal Batch Culture Fermentation, Protozoal Count, and Nutrient Degradability

After 24 h of incubation, all flasks were placed on ice to stop the microbial fermentation. Values of pH were determined by a portable pH meter (the same model that was used to measure the pH of the clays). The ammonia concentrations were determined calorimetrically using a commercial kit (Biodiagnostic kits, Giza, Egypt). The concentrations of SCFAs were measured according to Palmquist and Conrad [18] and adapted by Soltan et al. [3] using gas chromatography (GC; Thermo TRACE 1300, Rodano, Milan, Italy) equipped with a capillary column (TRFFAP 30 m × 0.53 mm ID × 0.5 μm film (thermo-part No: 260N225 P). The GC separation details have been reported by Salama et al. [16]. Protozoa were counted microscopy following the method described by Dehority et al. [19] using Neubauer improved bright-line hemacytometer counting chamber (Paul Marienfeld GmbH & Co. KG., Baden-Württemberg, Germany).

To determine the truly degraded organic matter (TDOM), the contents of the flasks were treated with the neutral detergent solution for three hour at 90 °C. The residuals non-degraded of the contents of the flasks were filtered in pre-weighed crucibles, washed with hot distilled water and acetone, dried, and allowed to be turned into ash. The TDOM was estimated by the difference between the incubated and non-degraded organic matter amounts, while the truly degraded neutral detergent fiber (TDNDF) was calculated by the difference between the amount of incubated NDF and the non-degraded NDF amounts [3]. The partitioning factor (PF) was calculated as the ratio of TDOM and net gas volume for 24 h [20].

### 2.4. Statistical Analysis

The *in vitro* assay was completed in one run (one day) for all experimental treatments. The actual statistical replications (*n* = 3) were the average of the analytical replicates (*n* = 6/inoculum). The experimental unit was the mean of the six analytical replicates formed one statistical replicate. All data were analyzed by one-way ANOVA using the MIXED procedure of SAS (SAS Institute Inc., Cary, NC, USA, version 9.0). Orthogonal contrast statements (contrast 1 and contrast 2) were designed to test each experimental parameter’s linear and quadratic responses to increasing concentrations (0, 0.05, and 0.5 g/kg feed substrate) of MNM_SDS_ and MNM_CETAB_, respectively. Comparisons among treatments were performed using Tukey’s test, the effects were declared significant at *p* ≤ 0.05, and the trends were accepted if *p* < 0.10.

## 3. Results

### 3.1. Physicochemical of NM, MNMsds, and MNM_CETAB_

Physicochemical characteristics of the experimental clays are shown in Table 2. Negligible variations in pH were detected among all clay products.

**Figure 1:**
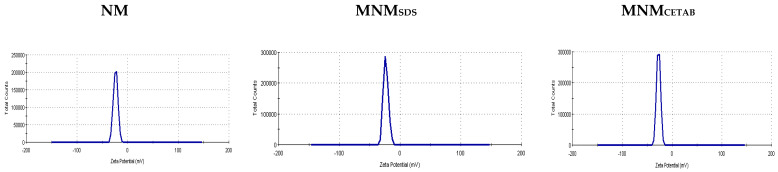
Zeta potential distribution for the experimental natural montmorillonite (NM), modified nano-montmorillonite by sodium dodecyl sulfate (MNM_SDS_), or cetyltrimethylammonium bromide (MNM_CETAB_).

The modification of NM either by CETAB or SDS resulted in a numerical reduction in the values of EC but enhanced CEC compared with the NM, MNM_CETAB_ had the lowest EC values, and MNM_SDS_ had the highest CEC values compared with other clays. The Zeta potential of NM clay was negative and became more negative after modifications by CETAB or SDS; MNM_CETAB_ had the highest zeta potential compared with the other clays (Figure 1).

**Figure 2:**
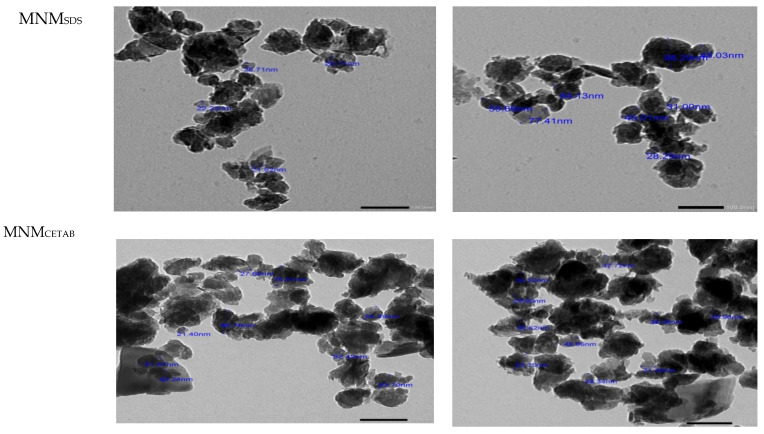
The nanoparticles size and shape transmission electron microscope (TEM) for the experimental modified nano-montmorillonite by sodium dodecyl sulfate (MNM_SDS_) or cetyltrimethylammonium bromide (MNM_CETAB_).

The average size of both MNM products was on the nano-scale; MNM_CETAB_ had the smallest nanoparticle size compared with the other clays. The TEM images of the size and size distribution of the experimental MNM particles are shown in Figure 2. TEM images confirmed the formation of nano nanoparticles of both MNM products; it also indicated the high quality of the synthesis method for producing similar nanoparticles. The images showed that most of the nanoparticles are within the 26.9–63.7 and 28.2–98.2 nm ranges for MNM_CETAB_ and MNM_SDS_, respectively.

**Figure 3:**
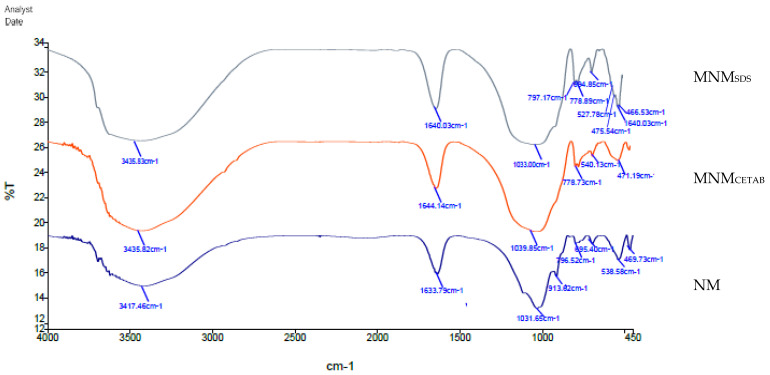
Fourier transform infrared spectroscopy (FTIR) spectra for the experimental natural montmorillonite (NM), modified nano-montmorillonite by sodium dodecyl sulfate (MNM_SDS_), or cetyltrimethylammonium bromide (MNM_CETAB_).

Figure 3 shows the results of the FTIR analysis to investigate the characteristics of MNM products compared with NM clay. In the high-frequency range, well-defined peaks (OH-group) were shifted from 3417 cm^−1^ in NM to higher frequencies at 3435 cm^−1^ in both MNM_CETAB_ and MNM_SDS_, and the bands frequency-shifted from 1633.7 cm^−1^ in NM to 1644 cm^−1^ in MNM_CETAB_ and 1640.03 cm^−1^ in MNM_SDS_. In the lower frequency range (750–1300 cm^−1^), a band at 778 cm^−1^ (attributed to the Si–O stretching vibrations) appeared only for both modified clays, while it was absent in NM. Three bands at 450 and 550 cm−1 corresponding to the bending mode of Si–O and Si–O–M bonds appeared in MNM_SDS_, while just two bands were observed in NM and MNM_CETAB_.

### 3.2. Effect of MNM on In Vitro Ruminal Batch Culture GP, CH_4_, Nutrient Degradability, and Partitioning Factor

Table 3 shows that NM treatment had the highest (*p* < 0.01) GP compared with antibiotic monensin and all MNM treatment except the low level of MNM_SDS_, while no differences were observed between the NM and the control. The contrast tests were significant for both MNM products. A linear decrease (*p* < 0.01) in GP values was observed by increasing the supplemental level of the modified clays. Similar CH_4_ reductions (*p* < 0.01) were observed by all MNM products and monensin treatments compared with the control. The high dose of both MNM treatments resulted in the highest reduction (*p* < 0.01) in CH_4_ production among all the experimental treatments. The contrast analysis showed that the decrease in CH_4_ was in a dose-dependent manner by MNM products; MNM_SDS_ reduced CH_4_ in linear (*p* < 0.01) and quadratic (*p* = 0.02) trends, while MNM_CETAB_ declined CH_4_ in a linear (*p* < 0.01) trend.

Monensin tended to reduce (*p* = 0.08) TDOM compared with MNM_CETAB_ treatments and decreased (*p* < 0.01) TDNDF compared with all MNM treatments. The contrast analysis showed that TDOM and TDNDF were not affected by MNM_SDS_ supplementation, while quadratic increases (*p* = 0.01) were recorded with the increasing doses of MNM_CETAB_ supplementation. All MNM treatments (except MNM_SDS_ low) enhanced (*p* < 0.01) the PF compared with the control. Increasing dosages of MNMsds showed linear increases (*p* < 0.01) in PF values, while MNM_CETAB_ showed both linear (*p* < 0.01) and quadratic (*p* = 0.01) increases in PF values.

### 3.3. Effect of MNM Supplementation on In Vitro Ruminal Batch Culture pH, NH_3_-N and SCFAs

The results of the effects of the experimental montmorillonite types on *in vitro* pH, NH_3_-N, and SCFAs are shown in Table 4. The high MNM_CETAB_ treatments increased (*p* < 0.01) ruminal pH compared with the control and monensin treatments. The contrast test showed that MNM_CETAB_ quadratically reduced (*p* = 0.01) *in vitro* ruminal pH while MNM_sds_ did not affect the pH values. The high doses of both MNM types, NM and monensin, decreased in (*p* < 0.01) NH_3_-N compared with the control. Both MNM types resulted in linear reductions (*p* < 0.01) in the NH_3_-N concentrations. The high MNM_SDS_ and all MNM_CETAB_ treatments increased (*p* < 0.01) the protozoal count compared with the monensin, and both MNM types linearly (*p* < 0.05) increased the protozoal count. The experimental treatments did not affect the total SCFAs concentrations, while modifications of molar proportions of individual SCFAs were observed. Increases in the acetate molar proportions and the acetate-to-propionate ratio were observed (*p* < 0.01) in the MNM treatments compared with monensin. Linear and quadratic increases (*p* < 0.01) in acetate molar proportions were marked by increasing levels of both MNM types. Monensin followed by MNM_CETAB_ treatment had the highest (*p* < 0.01) propionate molar proportions compared with other treatments. Treatments with MNM_SDS_ and MNM_CETAB_ had increased (*p* < 0.05) the propionate molar proportions linearly and quadratically. All MNM treatments and monensin presented similar reductions (*p* < 0.01) in butyrate compared with NM and control treatments. Linear and quadratic declines (*p* < 0.05) in butyrate were observed in MNM treatments. All of the experimental feed additives reduced isovalerate compared with the control (*p* < 0.01), while the high MNM_CETAB_ treatment had higher (*p* = 0.05) isobutyrate than monensin. Linear and quadratic decreases (*p* < 0.01) were observed by both MNM types, while MNM_CETAB_ presented quadratic increase (*p* = 0.03) in isobutyrate molar proportions.

## 4. Discussion

Natural montmorillonite is a 2:1 phyllosilicate clay and has a unit crystal lattice formed by one alumina octahedral sheet sandwiched between two silica tetrahedral sheets; its interlayer contains water molecules and inorganic cations [4]. Due to this unique form, NM has a high CEC and surface area compared with other 1:1 clays. The mechanical grinding and modification of the natural montmorillonite can lead to the interlayer’s collapse and can affect the swelling capacity and surface charge of the modified clays [4]. In this work, the physicochemical properties of the resultant MNM products were affected by the used surfactants compared with NM. The decrease in EC after CETAB and SDS modifications indicated that few electrons could move from the valence band to the conduction band [4]. Electrical conductivity is an indicator of salinity to measure a substrate’s ability to allow for the transport of electric charges; thus, the EC of clays may affect ruminal passage rate of the digesta, dilution of feed particles, and microbial degradability [21].

A high CEC was observed for the experimental NM (77.5 mmol/100 g), which became higher by CETAB and SDS modifications. This high CEC of both MNM types can indicate the high number of metal hydrolysates and ions that can be intercalated into the clay interlayer space [22], which in turn improves the clay activity compared with NM. This can be indicated by the frequency shifts and intensity of the hydroxyl H–O–H bond detected by FTIR analysis in the MNM clays compared to NM. Additionally, at the medium frequency range, a new band related to Si–O–Si bond [4] appeared only for both MNM clays, while it was absent in NM. These frequency shifts indicate the higher hydrophobicity of the resultant MNM clays than NM [22]. The most likely explanation for the differences between MNM_CETAB_ and MNM_SDS_ in CEC is the way in which the experimental surfactants bonded the clay interlayer space, which affected the structure and surface affinity of the resultant MNM products. Anionic surfactants as SDS have weaker interactions with the montmorillonite interlayer than cationic surfactants as CETAB [8]. Anions (SO_3_^−^) of SDS can be adsorbed on the edges of montmorillonite and compensated for its positive charges [8], while cations (NH_4_+) of CETAB can be adsorbed on the flat surface of the clay. This partly explains the higher negative charge of MNM_CETAB_ than MNM_SDS_.

Three bands at the low-frequency range corresponding to the bending mode of Si–O and Si–O–M bonds [4] appeared by FTIR analysis in MNM_SDS_, while just two bands were observed in MNM_CETAB_. This result could be due to the functional sharing group of SDS with the structure R-O-SO_3_−. The increase of negative charges of MNM_CETAB_ might be favorable to enhance its affinity with cationic matters, which might improve the adsorption capacity. These results confirm our first hypothesis that the physicochemical properties of MNM can be enhanced by the mechanical nano grinding and modification of NM. Thus, we evaluated their effects on rumen fermentation properties.

Rumen microbial fermentation is associated with the formation of greenhouse gasses (mainly CO_2_ and CH_4_). Montmorillonite is a potential adsorbent to capture CO_2_ through a reaction between CO_2_ molecules and its interlayer –OH groups by forming –HCO_3_− species, which in turn can react with other interlayer cations [23]. The reduction in GP caused by MNM types (especially MNM_CETAB_) may suggest that MNM had a higher absorbance capacity to capture CO_2_ than the NM. The literature reported that the modified montmorillonite has a higher CO_2_ reversible retention capacity than the NM due to increases in hydrophobic surface, interlayer spacing, and intercalation of organic cations between the base –OH sites of the clay and the CO_2_ molecules [23]. High CEC, shifts of the frequency and intensity of the hydroxyl H–O–H bonds detected by FTIR analysis, and increased negative charge of the MNM clays compared with NM may enhance the absorptive efficiency of MNM to capture CO_2_. It may be speculated that the CO_2_ adsorption is also varied by the solvated cations situated in the MNM interlayer spacing; hence, MNM_CETAB_ was the most effective clay to reduce GP.

Reductions in GP and CH_4_ were consistent with enhancements in protozoal count and degradability of OM and NDF by MNM types_,_ while this phenomenon did not appear after monensin treatment in the present study, in which CH_4_ inhibition by monensin was consistent with adverse effects on nutrient degradability and protozoal count. These results suggested that monensin had a different CH_4_ reduction mechanism from that of MNM. The antibacterial activity of sodium monensin against H_2_ producing bacteria (including methanogens and cellulolytic bacteria) arises from disrupting the cell membranes through the ion transport of H+/K+ and Na+/H+ cations [1,24]. Monensin is also known for the inhibition effects of ruminal fungi and protozoa, which contribute to fiber degradation [1,3]; thus, it partly explained the decreased TDOM combined with CH_4_ reduction by monensin therein. On the other hand, enhancing the protozoal count, TDOM, and TDNDF by MNM would promote H+ production. Hydrogen is the major intermediary metabolite in the ruminal degradation of NDF and OM that Archaea mainly use to reduce CO_2_ into CH_4_. Thus theoretically, enhancing the OM and NDF degradability promotes CH_4_ formation [1,25]. Therefore, CH_4_ reduction caused by MNM would indicate that it may bind not only CO_2_ but also H+. The increase in the intensity on the absorption bands of the OH group detected by FTIR analysis rather than the high negative charge zeta potential of the experimental MNM_CETAB_ would indicate the increased ability to bind the acidic H+. Increases in ruminal *in vitro* batch culture pH observed by MNM_CETAB_ may confirm such speculation, which in turn was favorable for microbial NDF degradation and may prevent ruminal acidosis.

Although the effect of MNM on the bacterial community was not evaluated (this has to be kept in consideration with MNM future studies), it can speculate that MNM has antibacterial effects against specific communities. However, both clay surfaces and bacterial cells have negatively charged sites; but the literature confirmed the ability of modified montmorillonite clays to bind them [5]. This is because of the presence of positively charged interlayer ions of the clay. In the current study, the changeable cations in MNM_SDS_ and MNM_CETAB_ in the clay edge or surface sites may affect the binding of rumen microbes to MNM surfaces through extracellular polysaccharides of the bacterial cell wall and, as a consequence, may affect the *in vitro* fermentation, including CH_4_ formation [5]. It seems that both MNM types can affect methanogenesis by possessing direct antibacterial activity since the protozoal counts and TDNDF were enhanced [25]. The literature confirmed the synergistic relationship between protozoa and methanogens. Protozoa can provide them with their end metabolites, including H_2_ [1]; thus, the protozoal count can indicate whether the treatments affected directly or indirectly the CH_4_ emission [1,3]. The anionic organo-sulfate surfactants (e.g., SDS) possess antibacterial and anti-inflammatory properties by sharing R–O–SO_3_^−^ functional groups [26]; thus, it may affect the antibacterial activity of the prepared MNM_SDS_. The more substantial reduction in CH_4_ caused by MNM_CETAB_ may be due to the quaternary positively charged ammonium group that can interact with Gram-positive bacterial cells, disrupt their cell membranes, and finally causes cell lysis [27]. Moreover, nano-clays have higher anti-methanogenic activity without adverse effects on the TDOM compared with their natural form [9]; this can partly explain the low effectiveness of NM to affect GP and CH_4_ compared with MNM in the current study.

Enhancements in PF values may also contribute to the CH_4_ reduction observed by MNM_CETAB_ [28]. Removing H+ from the rumen ecosystem is known to increase ruminal pH and to stimulate ruminal microbial activity; thus, when CH_4_ decreases, H+ may be used for producing SCFAs to ensure optimal ATP yield for the microbial mass production [2]. Increasing ruminal pH may increase protein solubility and generate branched-chain volatile fatty acids (BCVFA) production as isovalerate and isobutyrate [29]. Thus, it partly explains the increase in isobutyrate molar proportions consistent with high protozoal numbers and PF by MNM_CETAB_ treatment. A puzzling finding of the current study was the decrease in the isovalerate molar proportion found by all clay treatments compared with the control. No clear explanation for this finding can be presented. Branched-chain volatile fatty acids (BCVFA) such as isovalerate and isobutyrate can be produced from leucine and valine degradation, respectively [30]. Consequently, rumen microbes utilize the produced BCVFA to promote protozoa and microbial protein synthesis [31,32]. Thus, it can be assumed that clay treatments may likely be incorporated differently into the rate of microbial degradation of these amino acids and/or BCVFA utilization. Apajalahti et al. [30] found that not all BCVFA produced are incorporated similarly to the microbial protein synthesis.

The typical mode of action to reduce CH_4_ emission by monensin has occurred in this study by enhancing the redirections of the SCFAs pattern towards more propionate molar proportions and by reducing the acetate-to-propionate ratio [24]. The declines in ruminal *in vitro* batch culture pH, protozoal abundance, and TDNDF caused by monensin were favorable conditions for propionate producers [33]. On the other side, the associative enhancements in ruminal *in vitro* batch culture pH, protozoal numbers, and TDNDF were favorable conditions to acetate producers [3]; thus, acetate proportions were enhanced by MNM clays. Monensin inhibits Gram-positive bacteria, which are involved in protein degradation [24]. Therefore, further indications that monensin reduced the diet protein degradation can be provided by low TDOM, BCVFA, and ammonia values. Theoretically, enhancing TDOM may increase ammonia production; thus, it seems that ammonia reduction caused by MNM treatments was not a result of inhibition of protein degradation. Even NM treatment poetically reduced ammonia concentration without affecting TDOM. These results could be related to the presence of the acidic functional groups of the montmorillonite rather than the clay pore structure, which can enhance ammonia capture capacity to the clay. This function might be improved after the SDS or CETAB modifications because of the increases in CEC and shifts in the hydroxyl H–O–H bonds in addition to the more negative charge of MNM clays. These results may confirm our second hypothesis that MNM clays can modify the *in vitro* microbial fermentation, including CH_4_ emission, and this effect was type- and dose-dependent.

## 5. Conclusions

Two different feed additives of MNM have been developed at the nanoscale using cationic (CETAB) and anionic (SDS) surfactants. The modification and the mechanical nano grinding enhanced the physicochemical properties of the natural montmorillonite clay. Both MNM types had lower EC and higher CEC values than the natural clay. The MNM_CETAB_ showed a more significant negative charge than the other clays. All MNM clays and monensin successfully reduced the *in vitro* ruminal batch culture CH_4_ production and ammonia concentration, while MNM_CETAB_ enhanced TDOM, TDNDF, and pH compared with monensin. The experimental feed additives differently modified the SCFAs pattern. All MNM clays increased the acetate molar proportions, while only monensin increased propionate molar proportions. Under the conditions of this study, clay modified by cationic surfactant was more efficient than the anionic surfactant to modify rumen fermentation properties. The MNM_CETAB_ supplemented at 0.5 g/kg can be used as a novel natural feed additive to reduce CH_4_ without adversely affecting rumen fermentation or fiber degradability. These results emphasized that MNM clays can modulate *in vitro* microbial fermentation patterns in different pathways from that of monensin.

## Figures and Tables

**Table 1:** Major ingredients and chemical composition of the experimental basal feed substrate based on dry matter (DM).

Item	Experimental Feed Substrate
	(g/kg DM)
Ingredients	
*Trifolium alexandrinum* clover	500
Ground maize	233
Wheat bran	157
Soybean meal	90
Calcium carbonate	10
Sodium chloride	5
Vitamins and minerals mixture ^1^	5
Chemical composition	
Organic matter	903
Crude protein	143
Neutral detergent fiber	395
Acid detergent fiber	194
Acid detergent lignin	49.9
Ether extract	32.1

^1^ Mineral and vitamin mixture (mg/Kg): zinc, 60 mg; manganese, 80 mg; iron, 35 mg; copper, 8 mg; selenium, 0.6 mg; vitamin D3, 2500 International Unit (IU); vitamin A, 12,000 IU; vitamin E, 20 IU; menadione, 1.3 mg; riboflavin, 5.5 mg; vitamin B12, 10 µg; vitamin B6, 3 mg; thiamine, 3 mg; folic acid, 1.0 mg; d-biotin, 50 µg; Ca-pantothenate, 1 mg; nicotinic acid, 50 mg and choline chloride, 600 mg.

**Table 2:** Physicochemical properties of the natural montmorillonite (NM), modified nano-montmorillonite (MNM) by sodium dodecyl sulfate (MNM_SDS_), or cetyltrimethylammonium bromide (MNM_CETAB_).

Items	NM	MNM	
		MNM_SDS_	MNM_CETAB_
pH	8.048	8.054	7.900
Electrical conductivity (ppm)	1408	960	230
Cation exchange capacity (meq/100 g)	77.5	117	81.9
Zeta potential (mV)	−23.3	−23.8	−27.4
Average particle size (nm)	741.6	59.8	45.7

Sodium dodecyl sulfate (SDS; NaC_12_H_25_SO_4_; molar mass = 288.38 g/mol), cetyltrimethylammonium bromide (CETAB; C_19_H_42_BrN; molar mass = 364.45 g/mol).

**Table 3:** Effects of monensin, natural montmorillonite (NM), and modified nano-montmorillonite (MNM) supplementation on *in vitro* ruminal batch culture gas production, methane emission, and partitioning factor.

Item	Treatments (T)							SEM	*p* Value				
	Control	Monensin	NM	MNM					T	Contrast 1		Contrast 2	
				MNM_SDS_		MNM_CETAB_							
				Low	High	Low	High			Linear	Quadratic	Linear	Quadratic
Gas production (mL/g DM)	113 ^ab^	99.2 ^c^	117 ^a^	109 ^abc^	102 ^bc^	102 ^bc^	97.7 ^c^	2.73	<0.01	<0.01	0.56	<0.01	0.30
Methane production													
(mL/g IOM)	27.7 ^a^	19.5 ^bc^	25.9 ^ab^	20.1 ^bc^	17.3 ^cd^	20.2 ^bc^	11.9 ^d^	3.34	<0.01	<0.01	0.02	<0.01	0.69
(mL/g TDOM)	43.8 ^a^	31.4 ^bc^	41.2 ^ab^	31.7 ^bc^	26.8 ^cd^	30.1 ^c^	17.4 ^d^	5.85	<0.01	<0.01	0.04	<0.01	0.84
Nutrient degradability													
TDOM	632 ^ab^	614 ^b^	628 ^ab^	646 ^ab^	645 ^ab^	672 ^a^	652 ^a^	12.1	0.08	0.28	0.49	0.10	0.01
TDNDF	183 ^bcd^	143 ^d^	153 ^cd^	213 ^abc^	211 ^abc^	271 ^a^	227 ^ab^	20.74	<0.01	0.29	0.49	0.10	0.01
Partitioning factor	2.80 ^cd^	3.13 ^abc^	2.69 ^d^	2.97 ^bcd^	3.17 ^ab^	3.33 ^a^	3.35 ^a^	0.07	<0.01	<0.01	0.84	<0.01	0.01

MNM_SDS_ and MNM_CETAB_ = MNM modified by sodium dodecyl sulfate and cetyltrimethylammonium bromide, respectively. Low and high = MNM supplemented at 0.05 and 0.5 g/kg DM feed substrate, respectively. SEM = standard error of the mean. Contrast: 1 = effects of control (0 supplementation g/kg DM) compared with MNM_SDS_ supplementations, and Contrast: 2 = effects of control (0 supplementation g/kg DM) compared with MNM_CETAB_ supplementations. IOM = incubated organic matter. TDOM = truly degraded organic matter. TDNDF = truly degraded neutral detergent fiber. ^a,b,c,d^ Means within a row without a common superscript letter differ significantly at *p* ≤ 0.05.

**Table 4:** Effects of monensin, natural montmorillonite (NM), and modified nano-montmorillonite (MNM) supplementations on *in vitro* ruminal batch culture pH, ammonia concentrations (NH3-N), total short-chain fatty acids (SCFAs) concentration (mM), and molar proportions of individual SCFAs (% of total SCFA).

Item	Treatments (T)							SEM	*p*-Value				
	Control	Monensin	NM	MNM					T	Contrast 1		Contrast 2	
				MNM_SDS_		MNM_CETAB_				Linear	Quadratic	Linear	Quadratic
				Low	High	Low	High						
pH	5.59 ^b^	5.62 ^b^	5.63 ^b^	5.65 ^ab^	5.68 ^ab^	5.67 ^ab^	5.77 ^a^	0.025	<0.01	0.13	0.51	0.13	0.01
NH_3_-N (mg/100 mL)	22.1 ^a^	16.3 ^b^	16.6 ^b^	17.5 ^ab^	14.5 ^b^	17.1 ^ab^	14.4 ^b^	1.02	<0.01	<0.01	0.69	<0.01	<0.01
Protozoa (10^5^/mL)	7.02 ^ab^	6.00 ^b^	8.05 ^ab^	8.40 ^ab^	9.25 ^a^	9.00 ^a^	9.31 ^a^	0.597	<0.01	0.03	0.74	0.02	0.28
SCFAs													
Total (mM)	104	105	98.6	107	106	102	103	2.5	0.51	0.62	0.51	0.73	0.41
Acetate, % of total	56.7 ^b^	56.4 ^b^	56.2 ^b^	61.1 ^a^	61.1 ^a^	60.5 ^a^	60.1 ^a^	0.58	<0.01	<0.01	<0.01	<0.01	<0.01
Propionate, % of total	17.7 ^cd^	24.1 ^a^	18.2 ^bcd^	18.2 ^bcd^	18.0 ^bcd^	19.0 ^b^	18.9 ^b^	0.21	<0.01	0.08	<0.01	<0.01	<0.01
Butyrate, % of total	17.9 ^a^	14.0 ^b^	19.3 ^a^	13.9 ^b^	14.1 ^b^	13.1 ^b^	13.4 ^b^	0.38	<0.01	<0.01	0.03	<0.01	0.01
Isobutyrate, % of total	1.77 ^ab^	1.33 ^b^	1.46 ^b^	1.43 ^b^	1.42 ^b^	2.47 ^ab^	2.58 ^a^	0.19	0.015	0.28	0.56	0.03	0.31
Valerate, % of total	1.56 ^ab^	1.08 ^c^	1.57 ^a^	1.59 ^a^	1.72 ^a^	1.24 ^c^	1.25 ^bc^	0.04	<0.01	0.07	0.47	<0.01	<0.01
Isovalerate, % of total	4.17 ^a^	2.93 ^d^	3.83 ^b^	3.55 ^c^	3.69 ^bc^	3.52 ^c^	3.71 ^bc^	0.09	<0.01	<0.01	<0.01	<0.01	<0.01
C2/C3	3.19 ^a^	2.33 ^b^	3.20 ^a^	3.32 ^a^	3.32 ^a^	3.18 ^a^	3.18 ^a^	0.02	<0.01	0.01	0.13	0.88	0.88

MNM_SDS_ and OMNM_CETAB_ = MNM modified by sodium dodecyl sulfate and cetyltrimethylammonium bromide, respectively. Low and high = MNM supplemented at 0.05 and 0.5 g/kg DM feed substrate, respectively. SEM = standard error of the mean, C_2_/C_3_ = acetate to propionate ratio. Contrast: 1 = effects of control (0 supplementation g/kg DM) compared with MNM_SDS_ supplementations, and Contrast: 2 = effects of control (0 supplementation g/kg DM) compared with MNM_CETAB_ supplementations. ^a,b,c,d^ Means within a row without a common superscript letter differ significantly at *p* ≤ 0.05.

## Data Availability

Not applicable.
